# mSphere of Influence: Uncovering New Ways To Control Multidrug Resistance by Dissecting Essential Cell Processes

**DOI:** 10.1128/mSphere.00648-19

**Published:** 2019-09-25

**Authors:** Ana L. Flores-Mireles

**Affiliations:** a Department of Biological Sciences, University of Notre Dame, Notre Dame, Indiana, USA

**Keywords:** multidrug resistant, pathogens, antibiotics, CRISPR, essential genes, cell wall, multidrug resistance, pathogen, treatments

## Abstract

Ana L. Flores-Mireles works in the fields of microbial pathogenesis and development of new therapeutics. In this mSphere of Influence article, she reflects on how the papers “Bacterial cell wall biogenesis is mediated by SEDS and PBP polymerase families functioning semi-autonomously” by H. Cho et al. (Nat Microbiol 1:16172, 2016, https://doi.org/10.1038/nmicrobiol.2016.172) and “A comprehensive, CRISPR-based functional analysis of essential genes in bacteria” by J. M. Peters et al. (Cell 165:1493–1506, 2016, https://doi.org/10.1016/j.cell.2016.05.003) made an impact on her approach to dissecting essential processes to understand microbial pathogenesis in catheter-associated urinary tract infections and generate an effective treatment with reduced likelihood of developing resistance.

## Abstract (precis)

Ana L. Flores-Mireles works in the fields of microbial pathogenesis and development of new therapeutics. In this mSphere of Influence article, she reflects on how the papers “Bacterial cell wall biogenesis is mediated by SEDS and PBP polymerase families functioning semi-autonomously” by H.

## COMMENTARY

The rapid emergence of multidrug-resistant (MDR) pathogens in hospital and outpatient settings is one of the biggest global health challenges of our time. The plasticity of the bacteria in adapting and evolving to survive antibiotic treatments is faster than our capacity to produce new antibiotics or therapies to treat infections caused by MDR pathogens. The bacterial cell wall is an important component for protection against environmental stress, including antimicrobial compounds. Inactivation of cell wall biosynthesis has been an attractive target and focus for antimicrobial development since the discovery of antibiotics. However, bacteria have evolved mechanisms to counterattack the antibiotics’ activity. Thus, to be able to develop effective treatment options, we need to have a comprehensive picture of all the factors and processes involved in conferring on bacteria the capability to withstand antibiotics. A giant leap toward this goal is presented in the paper “Bacterial cell wall biogenesis is mediated by SEDS and PBP polymerase families functioning semi-autonomously” by Dr. Bernhardt’s group, where they reported the discovery of SEDS proteins, a new family of cell wall synthesizers (1). Before this work, it was assumed that cell wall assembly was done by the class A penicillin-binding proteins (aPBPs), the targets of penicillin-like drugs. Dr. Bernhardt’s group found that SEDS and aPBP systems are semiautonomous and that both are needed for full cell wall synthesis—completely changing the accepted paradigm in the field. This breakthrough has advanced our understanding of cell wall assembly, reshaping our view and opening new opportunities to develop efficient combinatorial therapies that can target both systems.

Antibiotic resistance in bacteria is usually the result of genes encoding inactivating enzymes or other functions that render the antimicrobial ineffective. In order to understand antibiotic resistance, a comprehensive list of these genes and their functions would be necessary. However, identification of new antibiotic targets in bacterial essential cell processes has been challenging since the tools available, such as transposon mutagenesis, generate disruptions in only nonessential genes. This limitation was overcome with the technique presented in the paper “A comprehensive, CRISPR-based functional analysis of essential genes in bacteria,” where the authors developed a CRISPR-based functional analysis of bacterial essential genes (2). This group created a library of essential gene knockdowns by using CRISPR interference. This technology enabled them to discover new direct antibiotic targets that otherwise were not possible. The development of CRISPR interference is a game changer, opening the possibilities to investigate and dissect essential gene networks involved in many bacterial behaviors, including antibiotic resistance, metabolic requirements during infection, virulence, immune evasion, host-microbe interaction, etc.

Traditionally, we focus on one factor to develop new therapies; however, these two studies have showed us that by understanding important players in essential bacterial networks, we can generate combinatorial therapies that will be more effective and in turn reduce the probability of resistance development. These approaches have expanded and influenced my perspective on my own research to obtain a comprehensive analysis of genes required for fitness, biofilm formation, and pathogenesis *in vivo*. This will be critical in our search for new ways to fight catheter-associated urinary tract infections, since multidrug resistance is a serious problem.
